# Derivation of Limbal Stem Cells from Human Adult Mesenchymal Stem Cells for the Treatment of Limbal Stem Cell Deficiency

**DOI:** 10.3390/ijms24032350

**Published:** 2023-01-25

**Authors:** Marta Cadenas-Martin, Francisco Arnalich-Montiel, Maria P De Miguel

**Affiliations:** 1 Cell Engineering Laboratory, La Paz University Hospital Health Research Institute, IdiPAZ, 28046 Madrid, Spain; 2 Ophthalmology Department, Ramón y Cajal University Hospital, Ramón y Cajal Health Research Institute, 28034 Madrid, Spain

**Keywords:** cornea, adipose-derived mesenchymal stem cells, differentiation, limbal stem cell deficiency

## Abstract

Approximately 10 million individuals have blindness due to limbal stem cell (LSCs) deficiency, one of the most challenging problems in ophthalmology. To replenish the LSC pool, an autologous extraocular cell source is appropriate, thereby avoiding the risk of immune rejection, the need for immunosuppression and the risk of damaging the contralateral eye. In recent years, adipose-derived mesenchymal stem cells (ADSCs) have been a key element in ocular regenerative medicine. In this study, we developed a protocol for deriving human LSCs from ADSCs compatible with the standard carrier human amniotic membrane, helping provide a stem cell pool capable of maintaining proper corneal epithelial homeostasis. The best protocol included an ectodermal induction step by culturing ADSCs with media containing fetal bovine serum, transforming growth factor-β inhibitor SB-505124, Wnt inhibitor IWP-2 and FGF2 for 7 days, followed by an LSC induction step of culture in modified supplemental hormonal epithelial medium supplemented with pigment epithelium-derived factor and keratinocyte growth factor for 10 additional days. The optimal differentiation efficiency was achieved when cells were cultured in this manner over vitronectin coating, resulting in up to 50% double-positive αp63/BMI-1 cells. The results of this project will benefit patients with LSC deficiency, aiding the restoration of vision.

## 1. Introduction

Corneal diseases are a major cause of vision loss, with over 6.3 million individuals going blind worldwide every year [1]. The World Health Organization estimates that approximately 285 million people have visual impairment, with approximately 5% caused by corneal disease [2]. Approximately 14.5 million people worldwide are unilaterally or bilaterally blind from corneal disease or injury, and approximately 2.0 million new cases are reported each year [3].

Currently, the standard treatment for these conditions consists of corneal transplantation (keratoplasty) from a human donor, which involves replacing the central part of the cornea. The success rate for this type of transplantation is approximately 80%; however, corneal diseases such as limbal stem cell deficiency (LSCD) do not benefit from these therapies, and there are few options for these patients (for a recent review, see [4]).

Clear vision requires an intact and properly stratified corneal epithelium, which originates from the cells in the ectoderm during embryogenesis [5]. Throughout life, these superficial corneal epithelial cells are constantly shed into the tear film [6] and are continuously replenished by limbal stem cells (LSCs), epithelial stem cells located at the basal layer of the limbus, the transition area between the cornea and the sclera [7]. More precisely, LSCs are compartmentalized within the limbus in the palisades of Vogt. These downward focal projections create the limbal epithelial crypts, which enable the interaction of LSCs with stromal cells and soluble growth factors. For example, ALDH1 upregulates when the limbal niche is damaged [8]. All of these special interactions, which help regulate LSC differentiation and self-renewal abilities, create the LSC niche [9,10,11].

LSCs differ from their differentiated progeny by their high holoclone formation capacity [12]; high proliferative potential in vitro or after activation in response to corneal injury; a slow cell cycle (a trait of stem cells’ quiescent state) [13]; their small size and high nucleus/cytoplasm ratio; the expression of a panel of putative stem cell markers, such as transporter protein ABCG2, transcription factors BMI-1 and C/EBPδ, cytoskeletal proteins—such as cytokeratins 5, 14, 15 and 19 and vimentin, and the cell adhesion molecules integrin α9 and β1 (for a detailed list, see [13] and references therein); and, more recently, novel putative LPC surface markers such as GPHA2, expressed on the surface of 0.4% of cultured limbal epithelial cells and predominantly expressed in the limbal crypts [14]. However, the most important LSC marker is the nuclear transcription factor p63α [4,12]. ΔNp63α has been shown to be more specific than other p63α isoforms as a marker for LSCs, and it is thought to be involved in the maintenance of LSCs’ proliferative potential [15].

As previously stated, penetrating or anterior lamellar keratoplasty (corneal transplantation) is not a valid treatment option for LSCD because the limbus is not removed from the donor cornea; hence, the LSC population is not replaced [16]. Therefore, ex vivo LSC expansion is currently the most widely used approach for treating LSCD. This technique is known as cultured limbal epithelial transplantation (CLET) [17] and has been commercialized as Holoclar (Ex Vivo Expanded Autologous Human Corneal Epithelial Cells Containing Stem Cells) [4,18]. The main advantage of this technique is the use of autologous cells, and thus, complete histocompatibility. However, CLET is only possible if enough healthy limbal tissue is available in the other eye (unilateral LSCD), given that most of these diseases are bilateral and, consequently, require allogenic tissue [19]. In addition, recurrent neovascularization and conjunctivalization often occur [20]. More recently, the clinical use of uncultured cells in simple limbal epithelial transplantation (SLET) has also been recently developed [21]. This technique has several advantages, such as its straightforward use, no delays and autologous nature; however, it presents similar drawbacks to cultured cells. Treating patients with bilateral LSCD or other corneal epithelium deficiencies remains challenging, and these complications have raised the need for alternative cell sources.

Several extraocular cell sources are being investigated for LSCD. Cultivated oral mucosa epithelial transplantation (COMET) is, to date, the most common choice for non-limbal autologous cells for treating LSCD and has the advantage of autologous use without the disadvantage of tissue scarcity. However, its main drawbacks are neo-angiogenesis following transplantation and methodological variations [22]. Furthermore, COMET cells are not stem cells and are terminally differentiated. This therapy is, therefore, only a short-term solution if the epithelium damage is due to LSCD because the therapy cannot replenish the stem cell population, and conjunctivalization often occurs [23]. Mesenchymal stem cells (MSCs) are the most promising extraocular cell source for treating LSCD, because adipose-derived stem cells (ADSCs) and bone marrow-derived MSCs show basal expression of corneal epithelial cell markers, such as ABCG2, p63, CK12 and CK76 [24], and have been shown to differentiate into epithelial cells [25,26]. Both MSC sources share the advantages of autologous use and tissue availability. MSCs also have colony-forming and holoclone-forming abilities, as do LSCs. A recent report showed less neovascularization in an animal LSCD model with undifferentiated ADSCs [26]. A recent proof-of-concept clinical trial, also with undifferentiated MSCs, showed promising results for treating this challenging disease (clinicaltrials.gov NCT01562002 [27]), demonstrating safety and effectiveness in restoring the corneal epithelial phenotype for treating and improving LSCD. The authors stated that “mesenchymal stem cell therapy deserves more preclinical investigational resources before the favorable results of this proof-of-concept trial could be transformed into the larger numbers of the multicenter trials that would provide stronger evidence”.

We recently published the first report showing that priming human ADSCs with LSC-specific culture medium can potentiate their therapeutic potential to promote corneal wound repair, restore transparency, decrease inflammation and modulate paracrine effector functions in an in vivo rat model of LSCD [28]. Nonetheless, these are differentiated cells and, therefore, can only aid LSCD in the short term.

Other extraocular cell sources being studied include hair follicle bulge-derived stem cells [29], conjunctiva-derived MSCs, human skin keratinocytes [30], Wharton’s jelly stem cells [31,32], dermal fibroblasts and human immature dental pulp stem cells (for outstanding reviews, see [4,33,34]); however, no further advantages with respect to the above-mentioned MSCs have been demonstrated. Embryonic stem cells (ESCs) and induced pluripotent stem cells (iPSCs) have also been widely studied as an alternative cell source for corneal epithelium reconstruction. In 2007, Ahmad et al. [35] first reported the differentiation of human stem cells into corneal epithelial-like cells, mimicking the conditions of the limbal niche by culturing human ESCs on a collagen type IV-coated substrate with limbal fibroblast-conditioned culture medium. The first reported use of human iPSCs for generating corneal epithelial-like cells was in 2012 [36,37], with more recently reported success suggesting clinical potential [38,39]. However, despite the theoretical advantage of iPSC-derived cells due to their higher differentiation potential than tissue-specific stem cells, major challenges need to be overcome before iPSC-derived cells can be used in actual therapy—challenges such as the links between pluripotency and associated tumorigenicity and the limited clinical experience in using iPSC-derived cells. The use of human ESCs also raises ethical issues.

Of all the extraocular cell sources for treating the corneal epithelium, only a couple [38,40] (both of which use iPSCs) have focused specifically on LSCs and p63 expression; i.e., the objective being to differentiate the extraocular cells to corneal epithelial cells and not to LSCs. Thus, their strategy for surface ocular regeneration appears to focus on producing terminally differentiated corneal epithelial cells, which would not be useful if the corneal epithelial damage was caused by LSC deficiency, given that the stem cell pool would not be replenished. 

The alternative we propose is to develop a protocol for deriving LSCs from an extraocular source, such as adult MSCs, and to provide the carriers and means to transplant them into the limbal niche, helping provide a stem cell pool capable of maintaining proper epithelial cell homeostasis and restoring vision in the long term.

## 2. Results

### 2.1. Differentiation into LSCs Is Achieved in Human ADSC Subpopulations Independently ofc-Kit Expression 

**Figure 1:**
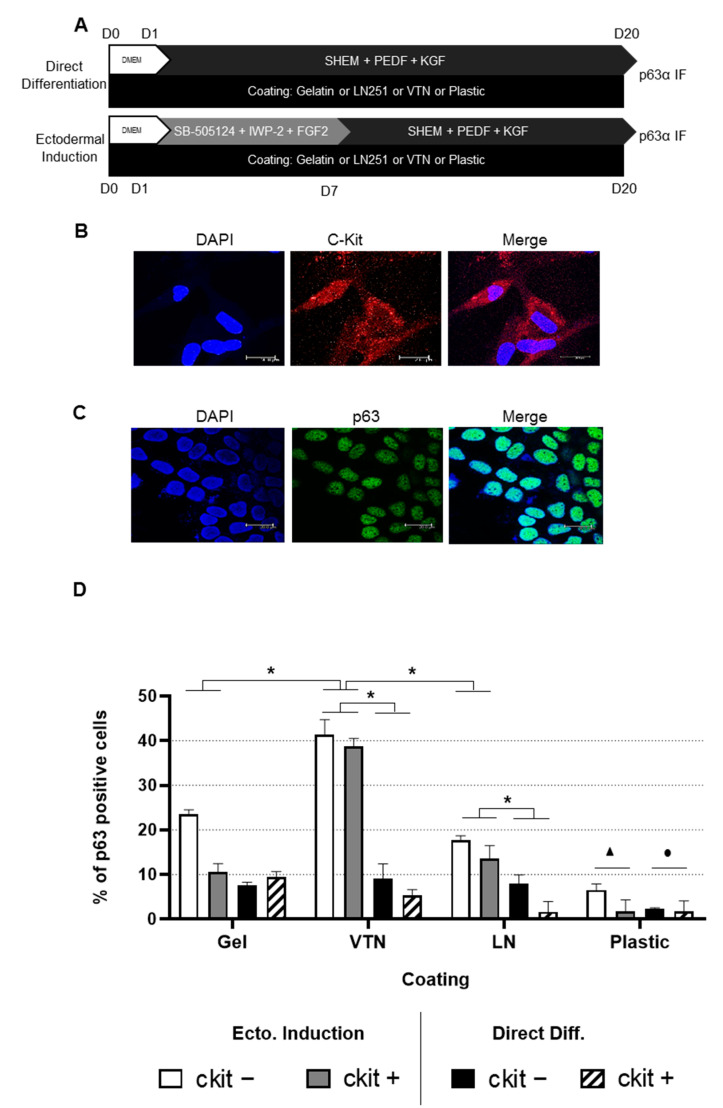
(**A**): **Schematic outline of the experimental design for hADSC to LSC differentiation**. The culture conditions, coating and duration for each stage are shown. The direct differentiation culture method consisted of culturing hADSCs in supplemental hormonal epithelial medium (SHEM) supplemented with two additional growth factors: pigment epithelium-derived factor (PEDF) and keratinocyte growth factor (KGF) for 20 days. The ectodermal induction method consisted of culturing hADSCs for 1 week with ectodermal induction medium before changing to the differentiation medium. Ectodermal induction medium consisted of Dulbecco’s Modified Eagle Medium supplemented with 15% fetal bovine serum, SB-505124, IWP-2 and FGF2. For each culture method, 4 different coatings were employed: gelatin (Gel), vitronectin (VTN), laminin (LN521) and plastic (no coating). After 20 days of culture, p63α expression was assessed by immunofluorescence staining. (**B**): **c-Kit (CD117) immunofluorescence**. Representative confocal microscopy images of c-Kit-positive hADSCs to confirm immunomagnetic separation; c-Kit in red and nuclei in blue; scale bars: 20 μm. (**C**): **p63α immunofluorescence.** Representative confocal microscopy images of HaCat cells labeled for p63α; p63α in green and nuclei in blue; scale bars: 20 μm. (**D**): **hADSC differentiation efficiency into LSCs**. Percentage of p63α-positive cells quantified (from 1 biological replicate with 2 technical replicates) from anti-p63α immunofluorescence at day 20 in differentiation medium, according to the coating condition and culture method employed. No statistical difference was found between c-Kit-positive and c-Kit-negative hADSCs. Previous ectodermal induction yielded a higher efficiency than directed differentiation. Regarding coating, the highest efficiency was achieved by coating with vitronectin, followed by gelatin and laminin, with plastic rendering the lowest. Error bars denote standard deviation. The Mann–Whitney U test was employed to assess differences between c-Kit-positive and c-Kit-negative cells; Student’s *t*-test was employed for assessing statistical significance between the culturing and coating method. *, ● and ▲ *p* < 0.05. ● indicates that under ectodermal induction all coatings were significantly different compared with plastic, and ▲ indicates that under direct differentiation only gelatin and vitronectin were significantly different compared with plastic.

**Figure 2:**
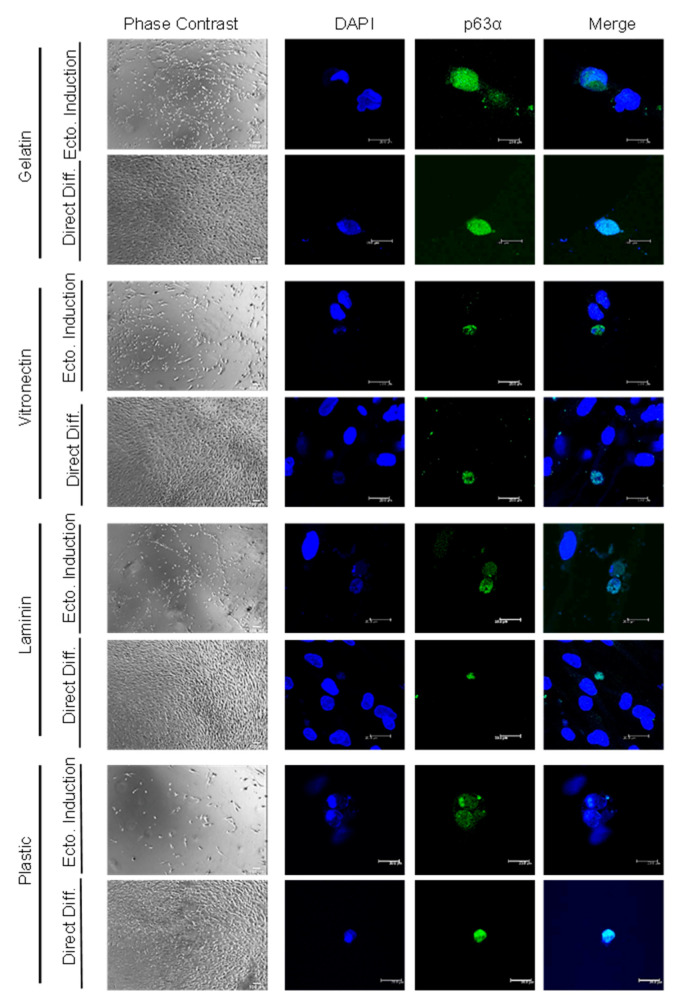
**p63α immunofluorescence of c-Kit-positive hADSC-derived LSCs**. Representative phase contrast and confocal microscopy images of c-Kit-positive hADSCs seeded on various coatings (gelatin, vitronectin, laminin and plastic) after employing 2 different culture methods: differentiation after ectodermal induction (Ecto. Induction) or direct differentiation (Direct Diff.); p63α in green and nuclei in blue; scale bar 200 μm (phase contrast) and 20 μm (immunofluorescence images).

**Figure 3:**
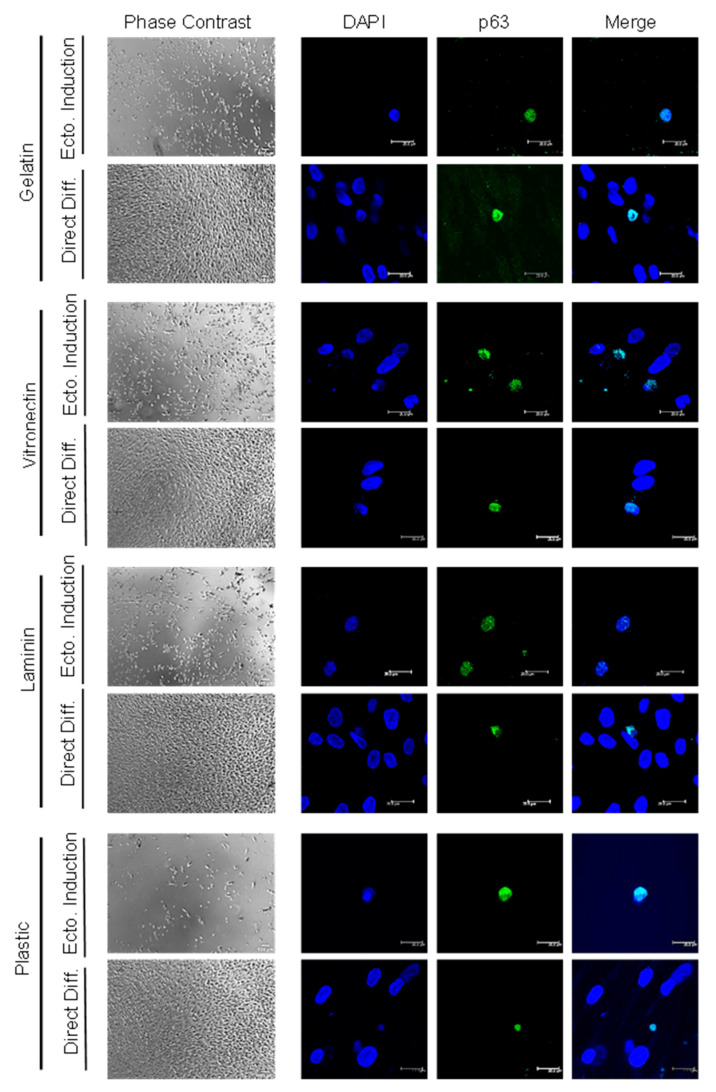
**p63α immunofluorescence of c-Kit-negative hADSC-derived LSCs.** Representative phase contrast and confocal microscopy images seeded on various coatings (gelatin, vitronectin, laminin and plastic) after employing 2 different culture methods: differentiation after ectodermal induction (Ecto. Induction) or direct differentiation (Direct Diff.); p63α in green and nuclei in blue; scale bar 200 μm (phase contrast) and 20 μm (confocal images).

C-Kit expression and, hence, the actual magnetic separation of c-Kit-positive and negative populations were confirmed by anti-c-Kit immunofluorescence (Figure 1B). Positive and negative c-Kit human ADSC (hADSC) cell differentiation efficiency was evaluated by immunofluorescence staining of the limbal stem cell marker p63α after culture in various coatings (gelatin, vitronectin, laminin and culture plastic/no coating), employing two differentiation methods (direct differentiation or differentiation after ectodermal induction) (Figure 1A). As a positive control for p63 expression, HaCat cells were employed, confirming 100% immunofluorescence detection (Figure 1C). HADSC differentiation into p63α-positive cells was achieved after 20 days, no matter the differentiation method (Figure 2 and Figure 3), in both the c-Kit positive and c-Kit negative subpopulations (Figure 2 and Figure 3, respectively). No statistically significant differences in differentiation efficiency were encountered between c-Kit-positive and c-Kit-negative cells, except over uncoated plastic (Figure 1D), as assessed by the Mann–Whitney U test.

### 2.2. High Differentiation Efficiency of hADSCs into LSCs Is Achieved by Previous Ectodermal Induction and Vitronectin Coating, Independent of c-Kit Expression 

When comparing the differentiation methods, prior ectodermal induction resulted in higher efficiency than directed differentiation in each coating (Figure 1D). In terms of coating, vitronectin achieved the highest efficiency, followed by gelatin and laminin, with plastic alone (no extracellular matrix coating) achieving the lowest (Figure 1D), both with previous ectodermal induction or directed differentiation, and in both the c-Kit-positive and c-Kit-negative subpopulations. Given that there were no significant differences between the c-Kit-positive and c-Kit-negative subpopulations, we performed subsequent studies with the unseparated ADSCs containing both subpopulations The best efficiency (reaching 40%) was thereby achieved by ectodermal induction, followed by differentiation, and on vitronectin coating, for a total of 20 days.

### 2.3. Differentiation over Amniotic Membrane Shows Morphological Changes, Accelerated Kinetics and Increased Cell Counts

**Figure 4:**
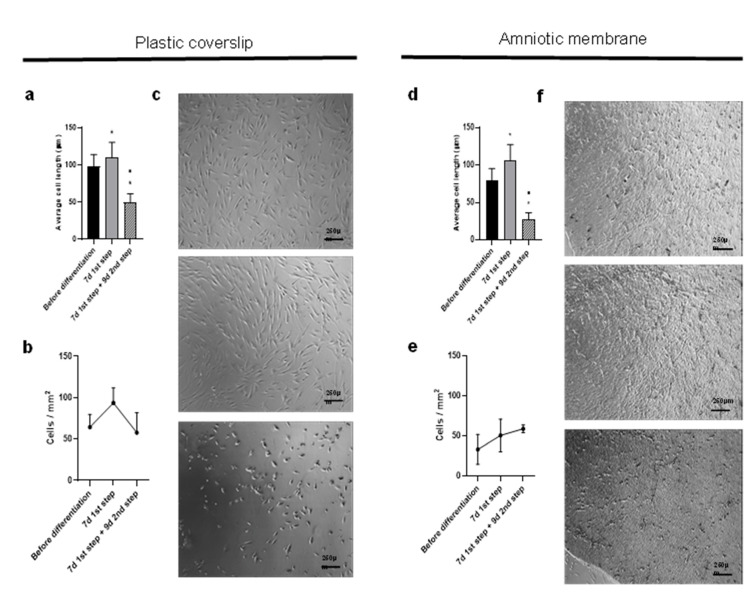
**Differentiation kinetics, morphology and cell numbers during differentiation of ADSCs into LSCs.** Mean cell length (in µm) (**a**,**d**), cell numbers per mm^2^ (**b**,**e**) and phase contrast images (**c**,**f**) before differentiation (top), after the first step (middle) and after the second step (bottom). All images feature a scale bar of 250 μm. Error bars denote standard deviation. We employed the Mann–Whitney U test to assess differences between c-Kit-positive and c-Kit-negative cells; Student’s *t*-test was employed to assess statistical significance. Data were pooled from 3 separate experiments comprising 3 wells each. Asterisks (*) indicate statistical significance with respect to before differentiation, and ■ indicates significance with respect to the first differentiation step at *p* ≤ 0.05.

We proceeded to differentiate ADSCs into LSCs on a human amniotic membrane (AM), a standard carrier, observing changes in cell morphology. To quantify our cell morphology observations, follow the differentiation kinetics and compare substrata as cell supports during differentiation, we quantified the length and number of ADSCs before differentiation, after 7 days of the first step and after 9 days of the second step, when the cells had already achieved the desired morphology. The cells both on plastic and AM significantly increased their mean length, as they differentiated into ectoderm on the first differentiation step and then reduced it (Figure 4a,d). The cells showed a progressive rounding up during the second differentiation step and reduced their final length to 50 µm on plastic and to almost 30 µm on AM.

We also observed changes in cell numbers. Over plastic, the tendency was for the cells to actively replicate during the first differentiation step and then decrease during the second step. However, when differentiation occurred over AM, the cell counts also increased during the second step (compare Figure 4b,e).

When ADSCs were seeded on AM, differentiation was faster than on culture plastic, and cells acquired the appropriate morphology in a total of 16 days, requiring only 9 days for the second differentiation step (Figure 4c,f).

### 2.4. The Percentage of ADSC-Derived LSCs Positive for LSC Markers Peaks at 17 Days of Differentiation

Given that there is no single specific LSC marker but rather several markers in the corneal limbal niche, we compared the differentiation efficiency between plastic and AM carriers coated with vitronectin by detecting the colocalization of nuclear p63α/BMI-1 and the expression of the cytoplasmic SSEA4 embryonic cell marker by immunofluorescence.

**Figure 5:**
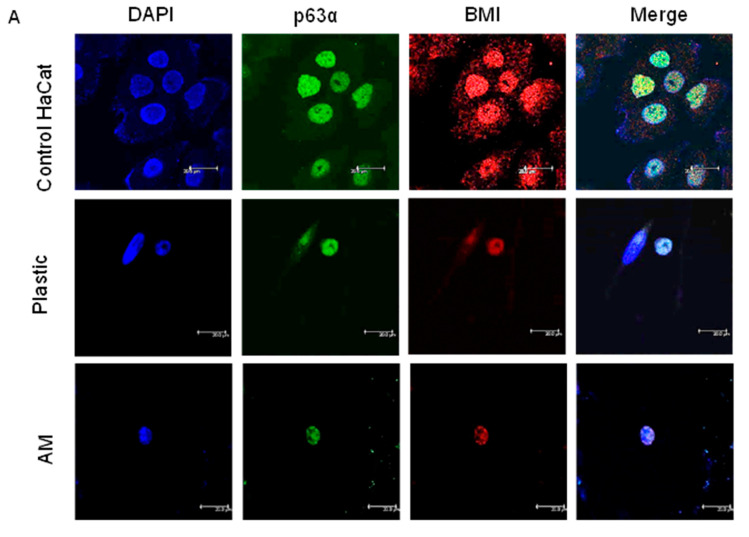

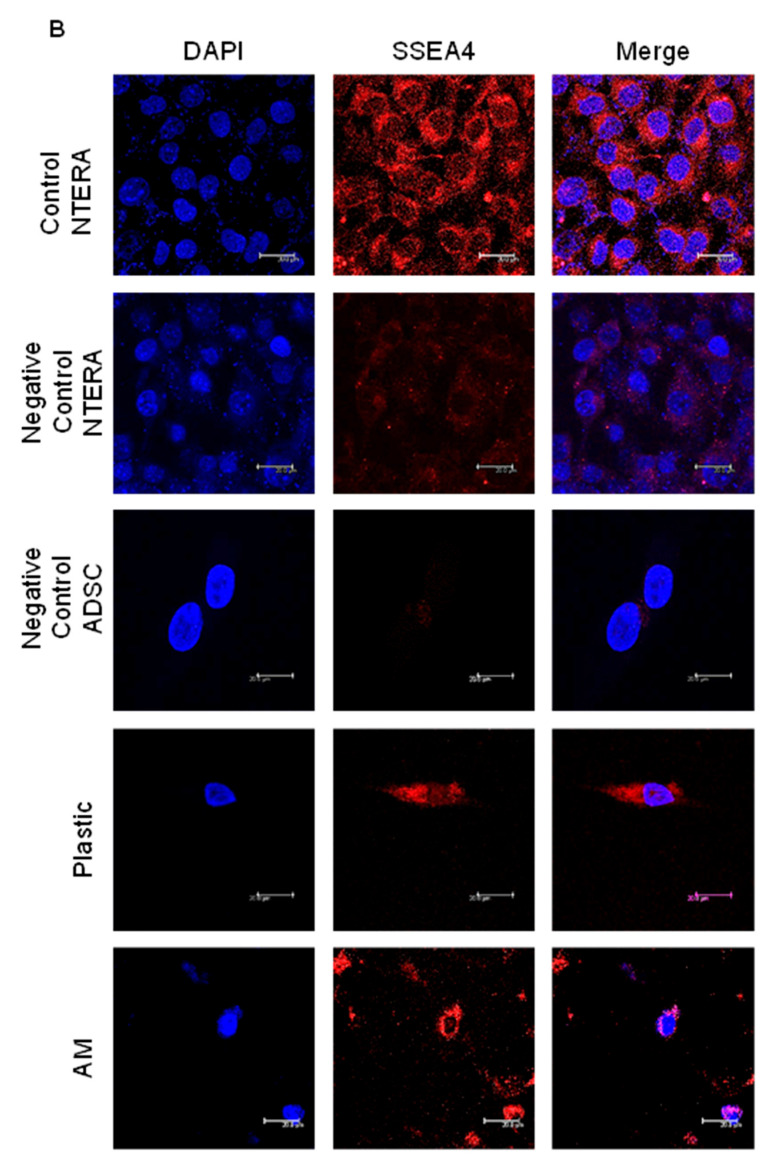
(**A**): **p63α/BMI immunofluorescence during LSC differentiation.** Confocal microscopy images showing double p63α/BMI immunofluorescence and DAPI staining. HaCat cells as positive control; LSCs from donor 2 on plastic coverslips or amniotic membrane carriers after 13 days at the second differentiation step. (**B**): **SSEA4 immunofluorescence during LSC differentiation.** Confocal microscopy images showing SSEA4 immunofluorescence and DAPI labeling. NTERA cells as positive control showing scattered cytoplasmic labeling; NTERA cells and native ADSCs as negative controls incubated with secondary antibody only showing no labeling. LSCs from donor 2 on plastic or on amniotic membrane carriers after 11 or 10 days at the second differentiation step, respectively. All images feature a scale bar of 20 μm.

In ADSC-derived LSCs at 10 days of the second step of differentiation, we found up to 53.3% positivity for both markers p63α and BMI-1 on the plastic coverslip (coated with vitronectin). Differences were encountered between donors, varying from 26.3% to 53.3% double positivity p63α/BMI-1 at 10 days of the second differentiation step on plastic coverslips. When the second step was 1 day longer (11 days), the ADSC-derived LSCs presented up to 43.4% positive cells, a percentage maintained at 13 days of the second differentiation step, at 41.4%. When cultured on AM (coated with vitronectin), the percentage of double-positive p63α/BMI cells at 13 days was 34.1% (Figure 5A). In conclusion, the optimal duration of the second differentiation step was 10 days.

Regarding the SSEA4 marker, after 10 days of culture at the second differentiation step, 52.9% of the LSCs derived from hADSCs on plastic (coated with vitronectin) were positive. When the second differentiation stage was 1 day longer (11 days), LSCs derived from hADSCs on plastic rendered 50.9% positivity for SSEA4. On AM (coated with vitronectin), the percentage of SSE4-positive cells at 13 days of culture at the second differentiation step was 44.5% (Figure 5B). SSEA4 labeling was scattered in the cytoplasm of control NTERA2 cells, whereas in ADSC-derived LSC it was localized in a more discrete section of the cytoplasm; this could be due to morphological differences between the control and studied cells, NTERA being large and spread and LSC being small and rounded with a higher nucleus-to-cytoplasm ratio. In conclusion, the optimal duration of the second differentiation step was 10 days, i.e., a total in vitro differentiation time of 17 days.

## 3. Discussion

One of the most challenging problems in clinical ophthalmology is the reconstruction of the ocular surface epithelium in patients with bilateral LSCD. To replenish the stem cell pool, an autologous extraocular cell source is appropriate for ex vivo culture, tissue engineering and transplantation, thereby avoiding the risk of allogenic immune rejection, the need for immunosuppression and the risk of damaging the contralateral eye [2]. In recent years, ADSCs have been a key element in ocular regenerative medicine because they can be obtained from abundant adipose tissue by a minimally invasive procedure, resulting in a high number of retrieved cells and with the capacity to differentiate into multiple cell lineages, including corneal epithelial cells [13,26]. To date, undifferentiated ADSCs and ADSCs primed towards the corneal epithelium have been transplanted [27,28]; however, the objective of replenishing the limbal niche, and thus, regenerating the corneal epithelium in the long term has not been achieved. 

In this study, we chose to examine the capacity of hADSC subpopulations (based on c-Kit expression) to transdifferentiate into LSCs. We obtained p63α-positive cells after 20 days with each method employed, in both the c-Kit-positive and c-Kit-negative subpopulations. Shorter times did not achieve proper transdifferentiation (not shown). Although previous studies have shown that c-Kit (CD117)-positive hADSCs showed a higher differentiation potential to specific cell lineages (adipogenic, pancreatogenic and hepatogenic [41]), in this case, c-Kit-negative cells rendered slightly higher p63α-positive numbers than the c-Kit-positive subpopulation, although there was no statistical significance. Despite a higher differential potential to specific lineages, those lineages tend to be of endodermal origin; c-Kit has, therefore, been proposed as an endodermal marker [42]. Given that the total c-Kit-positive cell population accounts for approximately 0.5% of the stromal vascular fraction [41], we chose to use the bulk population for subsequent studies.

We tested various coating proteins (gelatin, vitronectin, laminin and culture plastic) to favor transdifferentiation. The lowest percentage of p63α-positive cells was always achieved when cells were cultured in culture plastic alone (4% with previous ectodermal induction and 2% with direct differentiation) compared with gelatin (17% and 8%, respectively) and laminin (16% and 5%, respectively), with vitronectin achieving the highest (40% and 7%, respectively). Previous ectodermal induction favors LSC differentiation; however, these differences were significant only with the vitronectin and laminin coating when compared with direct differentiation. Vitronectin, laminin and, to some extent, gelatin (denatured collagen type IV) mimic the LSC extracellular matrix composition, aiding the differentiation process. The underlying basement membrane of the limbal stem cell niche where LSCs reside appears to be a key element in certain cellular activities, such as cell growth, proliferation, migration and differentiation [43]. For example, laminin and vitronectin are present in the stem cell niche but absent in the central cornea [44]. Furthermore, human LSCs expanded on laminin [45] or vitronectin-coated plates have the ability to generate larger holoclone-like colonies and present higher colony-forming efficiency [46]. It is, therefore, not surprising that the best result was achieved with differentiation after ectodermal induction on vitronectin coating, which yielded up to 40% p63α-positive cells. 

The comparison of the two transdifferentiation methods (direct differentiation and differentiation after ectodermal induction) clearly tilted towards the previous ectodermal induction. During embryogenesis, corneal epithelium and LSCs originate from the surface ectoderm [5]. Although many signaling pathways remain unidentified, it is known that blocking transforming growth factor (TGF)-β and Wnt signaling routes is required for the development of ocular surface ectoderm [47,48]. Mimicking these biochemical cues that occur during the developmental process could explain why a prior ectodermal induction before direct differentiation yields a higher percentage of p63α-positive cells when compared with direct differentiation only. Inhibitor of Wnt production 2 (IWP-2), which is present in the ectodermal induction medium, could function as an inhibitor of the Wnt pathway by inhibiting an enzyme called porcupine, which is involved in the post-translational modifications related to Wnt secretion [49]. In addition, SB-505124 (also present in the ectodermal induction medium) selectively inhibits the TGF-β pathway by efficiently blocking TGF-β type I receptors [50], aiding in the increased efficiency possibly by preventing the epithelial-to-mesenchymal transition [51]. 

The ectodermal induction medium used in this study was based on a protocol developed by Mikhailova et al. [52], comprising human iPSCs and a coating made of a mixture of collagen IV and laminin; after ectodermal induction, hiPSCs were cultured in CnT-30 medium (chemically defined commercial medium for corneal epithelium). The authors reported up to 81% p63α-expressing cells. More recently, a similar method rendered clinically relevant LSCs [53]. In addition to the protocol differences, hiPSCs have a higher differentiation potential than hADSCs, which could explain why the authors could obtain higher numbers of p63α-positive cells. However, the links between pluripotency and tumorigenicity associated with hiPSCs raise important safety concerns; thus, their clinical use is limited when compared with hADSCs [31]. In addition, hiPSC technology is still an emerging, exorbitantly expensive, time-consuming and difficult technique in many laboratories, despite outstanding recent advances [39,54]. Thus, the possibility of using hADSCs of autologous origin, with a simple, relatively cheap and chemically defined medium and differentiation protocol is an advance in their clinical use in LSCD. 

According to our results, the addition of PEDF, which promotes LSC proliferation [55], and KGF, which increases LSC growth by upregulating ΔNp63α [15] to SHEM medium, promoted p63α expression in hADSCs. This finding is relevant because SHEM medium has already been used to differentiate hADSCs into corneal epithelial cells [31]. However, p63α expression in hADSCs cultured in SHEM medium alone decreased over time, as assessed by a quantitative polymerase chain reaction (qPCR) [28]. This could be due to the epidermal growth factor (EGF) contained in SHEM, which has been shown to promote corneal epithelial restoration, possibly promoting LSC differentiation (for a review, see [56]). Our data show that the addition of only two growth factors (PEDF and KGF) allows for further de-differentiation of corneal epithelial cells to LSCs, reaching up to 53% p63α-positive cells. Fortunately, the cell cultures of actual LSCs used for LSC transplantation containing more than 3% of p63α-positive cells are associated with a 78% success rate, whereas transplants containing fewer than 3% p63α-positive cells were successful in only 11% of patients with LSCD [57]. hADSCs express baseline p63α levels when cultured in noninductive medium. However, the baseline p63α levels of hADSCs are so low (100-fold lower than in LSCs) that they are only detectable using qPCR and not by Western blot or immunofluorescence [24]. Our culture methods upregulated p63α protein levels; thus, we could detect its expression by immunofluorescent staining. In this regard, our outstanding percentage of p63α-positive cells hints at a high clinical potential.

To establish whether there had been a proper differentiation, we also performed immunofluorescence for BMI-1 on ADSC-derived LSCs, given that there is no consensus LSC stem cell panel marker, because the broadly accepted p63α [12] is not present in every cell of the limbal niche. Mitotically quiescent LSCs co-express BMI-1 and p63α under normal homeostasis [13,58]. This duo identifies human limbal holoclones and is a part of the genetic program maintaining stem cell identity [58]; regardless, the percentage of p63α/BMI-1-positive cells in the LSC niche is unknown. We obtained p63α/BMI-1 double-positive LSCs up to 53.3%. We propose this duo to be sufficient for LSC identification, although a larger set of markers could further identify LSCs (K5, K14, K15, ABCG2, ABCG5, Pax6) [34,53]. 

SSEA4 has not only been described as a stem cell marker, but also as a marker of more differentiated epithelial cells [59]. One article reported 12% of SSEA4-positive ADSCs [60], whereas another study claimed no expression [61]. The high percentage of SSEA4-positive cells we achieved could come from LSCs, more differentiated corneal epithelial cells or ADSCs. Even if the presence of ADSCs prevents the LSC population from being pure, the ADSCs could also be beneficial, given that the expression of epithelial markers is one of the characteristics that make ADSCs suitable as a source for LSCD treatment [24]. To differentiate these populations, the detection of other markers could be an advantage. For example, BMI-1 has not been reported in the literature or in the Human Protein Atlas to be expressed in ADSCs or epithelial cells; thus, double-positive p63α/BMI percentages are the most reliable method for identifying LSCs, as discussed above.

In any case, given that it is still unclear which markers define the true LSC in vivo, functional studies with various proportions of these populations should be performed to ascertain the actual identity of the LSCs and which cells show the highest regeneration potential.

The number of cells during differentiation varied between donors. In two-thirds of the experiments on plastic coverslips, the cells underwent an initial increase in number and a subsequent decrease in number similar to plated ones. The initial increase in replication could be due to the higher proportion of fetal bovine serum (FBS) in the Mikhailova medium (15%) than in the differentiation medium (2%), and the subsequent decrease in cells could be due to them acquiring a slow cell cycle, which is a stem cell characteristic [13]. The other third of the experiments showed a significant increase after second step differentiation. These results could be explained by differences between donors. The three donors differed in age (55, 44 and 50 years old), fertile life stage (postmenopausal, premenopausal and postmenopausal, respectively) and body mass index (26.4, overweight; 24, normal weight; 31.8, overweight, respectively). It has been shown that older donors with a higher body mass index provide more senescent ADSCs (as occurred with donor 1, where the number of cells decreased compared with other experiments) and have less differentiation capacity, less proliferation capacity and longer cells (as occurred in our ADSCs from donors 1 and 3 with respect to donor 2) [62], as well as the lack of estrogen, reducing cell viability. This all correlates with our results, given that ADSCs from donor 2 (the youngest, premenopausal and with normal weight) presented the highest proliferation after the second step of differentiation, followed by donor 3. Interestingly, the percentage of p63α/BMI-1-positive LSCs also differed between donors: that from donor 1 (26.3%) was less than that from donor 2 (53.3%), even if cultured under the same substratum and differentiation time. Given that ADSC-derived LSCs from donor 2 were the most proliferative, they were chosen to test the differentiation process over AM. 

The process of ADSC to LSC differentiation was accompanied by a progressive shortening of cell length and rounding up of cell morphology, accompanied by an increase in the nucleus-to-cytoplasm ratio (two LSC characteristics) compared with native hADSC spindle-shaped morphology and low nucleus-to-cytoplasm ratio [34]. This change was both faster and more profound on AM, with rounder and smaller cells, hence, becoming similar to actual limbal cells (which are rounded cells of approximately 12 µm in diameter). This is possibly due to the collagen composition of AM mimicking the LSC niche [63,64], which could alter the stiffness of the niche, promoting LSC development (for a thorough review, see [34]). Several interesting artificial carriers are currently being developed as substitutes for AM [4,28,34,65,66,67,68,69], together with other transplantation routes already reaching clinical trials (NCT04224207, NCT02144103, NCT03011541, NCT02325843, NCT01808378, NCT04484402, NCT03967275, and NCT03237442 (for a thorough review see [70])).

In conclusion, we have developed a rapid and inexpensive two-step method with a previous ectodermal differentiation step and a specific growth-factor combination over a vitronectin protein coating surface, which allows for the in vitro differentiation of ADSCs into LSCs. This transdifferentiation protocol is optimally achieved in 17 days, obtaining an LSC population of a mean 40% of p63α/BMI and 45% of SSEA4-positive cells. As the differentiation process moves forward, cells first elongate and then become round, and the numbers slightly increase. Our data help provide a stem cell pool compatible with amniotic membrane transplantation, which is capable of maintaining proper corneal epithelial homeostasis. Further in vivo studies are needed to ascertain whether these cells are capable of restoring the LSC niche in LSCD preclinical models.

## 4. Materials and Methods

Human adult ADSCs from the La Paz University Hospital Biobank were used for transdifferentiation, fulfilling EC regulation 1394/2007. These cells were obtained within this project, given that they had already been harvested, with informed consent signed from healthy volunteers, and biobanked with consent for subsequent studies. The donor data were anonymized by the La Paz University Hospital Biobank. We employed cells from 3 different donors (age 55, 44 and 50 years, body mass index of 26.4 (overweight), 24 (normal weight) and 31.8 (overweight), respectively).

### 4.1. Immunomagnetic Separation of hADSC Subpopulations

Previous studies by our group have shown that c-Kit (CD117)-positive hADSCs have a higher differentiation potential to specific cell lineages [41]. To assess whether c-Kit-positive hADSCs have a higher differentiation efficiency into LSCs, we first selected hADSCs by magnetic separation using anti-c-Kit antibody conjugated MicroBeads (Miltenyi), following the manufacturer’s instructions. 

### 4.2. In Vitro Directed Transdifferentiation into Limbal Stem Cells

We first used a direct transdifferentiation approach, employing a basic differentiation medium consisting of SHEM plus additional growth factors [28]. SHEM medium is composed of Dulbecco’s Modified Eagle Medium (DMEM)/Ham’s F12 (2:1), 862 mg/L of GlutaMAX (Gibco, Waltham, MA, USA), 110 mg/L of pyruvate (Gibco), 2% FBS and 1% penicillin/streptomycin, supplemented with 5 μg/mL of insulin (Humalog, Indianapolis, IN, USA); 10 ng/mL of human EGF (AF-100-15, PeproTech, Rocky Hill, NJ, USA), 0.18 mM of adenine (A2786, Merck, Darmstadt, Germany; 0.4 μg/mL of hydrocortisone (H0888, Merck), 2 nM of 3,3′,5-triiodo-L-thyronine (T6397, Sigma Aldrich) and 0.5% dimethyl sulfoxide (EMSURE, Chicago, IL, USA). We previously used SHEM to differentiate hADSCs into corneal epithelial cells [28]. To further (de)differentiate these cells into LSCs, we added growth factors consisting of 4 nM of PEDF (130-13, PeproTech) and 20 ng/mL of KGF (100-19, PeproTech), based on published papers showing that PEDF promotes the self-renewal of LSCs and KGF promotes LSC growth by upregulating ΔNp63α [15,55] (Figure 1A). To increase transdifferentiation efficiency, we performed a 2-step differentiation protocol consisting of a previous ectodermal induction step, parallel to what we had previously successfully accomplished for corneal endothelial cells [71]. We incubated the ADSCs according to a previously published protocol for differentiating hiPSCs into LSCs [52], consisting of DMEM, 862 mg/L of GlutaMAX, 110 mg/L of pyruvate, 15% FBS, 1% penicillin/streptomycin supplemented with 10 μM of TGF-β inhibitor SB-505124 (S4696 Merck), 10 μM of Wnt inhibitor IWP-2 (I0536, Merck Millipore) and 50 ng/mL of FGF2 (01-A01110, ORF Genetics, Kopavogur, Iceland). After 1 week, the ectodermal induction medium was replaced by the modified SHEM differentiation medium just as in the direct differentiation protocol. Thus, 2 differentiation protocols were tested, one by direct differentiation and another by a previous ectodermal differentiation step (Figure 1A). The cells were cultured at 37 °C in 5% CO_2_ in the differentiation medium for another 13 days, in which the medium was changed every other day (Figure 1A).

We tested various extracellular matrix proteins to increase the de-differentiation efficiency, given that the elastic modulus and substrate composition have been shown to affect LSCs [9]. We coated the culture plates with 1% gelatin type A (denatured collagen IV) (G1890,Merck), 0.5 μg/cm^2^ of laminin 521 (LN521, BioLamina, Stockholm, Sweden), 0.75 μg/cm^2^ of vitronectin (A14700, Gibco) or culture plastic alone (uncoated) (Figure 1A).

### 4.3. Limbal Stem Cell Marker Characterization

Given that there is no unique LSC marker, a set of markers was used to assess the transdifferentiation of ADSCs into LSCs by using immunofluorescence and confocal microscopy: ΔNp63α (Cell Signaling Technology, Danvers, MA, USA, 1:100), BMI-1 (Santa Cruz Biotechnology, Dallas, USA, 1:60), and the stem cell marker SSEA4 (R&D Systems, Minneapolis, MN, USA, 1:100).

### 4.4. Amniotic Membrane Preparation

Given that human amniotic membrane is the standard carrier for CLET, we employed human amniotic membranes not suitable for transplantation. These were obtained from the tissue biobank Banc de Sang i Teixits Barcelona (Barcelona, Spain). Data from the donor was anonymized at the biobank. AM was preserved in glycerol and frozen at −80 °C until use. Before use, the AM was washed 4 times for 15 min and scraped to decellularize both sides.

### 4.5. Statistics

We repeated all the cell culture experiments at least 3 times, comprising at least 3 internal replicates per experiment. We employed mean and standard deviation to describe the continuous variables. We performed statistical comparisons between groups using a Student’s *t*-test derived from a mixed model analysis of variance if normality assumptions were satisfied. We employed the mixed model to account for the correlation among multiple measures of an outcome on the same subject at different time points and/or different corneal zones. In case normality assumptions were not satisfied, we determined the significance of observed differences between the study groups using the Kruskal–Wallis one-way analysis of variance by rank (K–W) and the Mann–Whitney U test (M–W). A value of *p* < 0.05 was considered to be statistically significant.

## Data Availability

No new data were created.
